# Checklist of the leaf-mining flies (Diptera, Agromyzidae) of Finland

**DOI:** 10.3897/zookeys.441.7586

**Published:** 2014-09-19

**Authors:** Jere Kahanpää

**Affiliations:** 1 Finnish Museum of Natural History, Zoology Unit, P.O. Box 17, FI–00014 University of Helsinki, Finland

**Keywords:** Checklist, Finland, Diptera, biodiversity, faunistics

## Abstract

A checklist of the Agromyzidae (Diptera) recorded from Finland is presented. 279 (or 280) species are currently known from the country. *Phytomyza linguae* Lundqvist, 1947 is recorded as new to Finland.

## Introduction

The Agromyzidae are called the leaf-miner or leaf-mining flies and not without reason, although a substantial fraction of the species feed as larvae on other parts of living plants. While Agromyzidae is traditionally placed in the superfamily Opomyzoidea, its exact relationships with other acalyptrate Diptera are poorly understood (see for example Winkler et al. 2010).

Two subfamilies are recognised within the leaf-mining flies: Agromyzinae and Phytomyzinae. Both are now recognised as natural groups (Dempewolf 2005, Scheffer et al. 2007). Unfortunately the genera are not as well defined: at least *Ophiomyia*, *Phytoliriomyza* and *Aulagromyza* are paraphyletic in DNA sequence analyses (see Scheffer et al. 2007). The same studies and Winkler et al. (2009) suggest that *Chromatomyia* consists of at least two separate groups of species within *Phytomyza*, and *Napomyza* (which is given a status of a subgenus of *Phytomyza* by Winkler et al. (2009)) also needs re-evaluation. For the purposes of this checklist, a conservative approach is adopted with *Chromatomyia* and *Napomyza* as separate genera.

Richard Frey was the first Finnish dipterist to study the agromyzid fauna of the country in detail (Frey 1937, 1946). L. Tiensuu and E. Thuneberg continued Frey’s work but most of their work remained unpublished until Nowakowski (1967) and Spencer (1976) reviewed the major Finnish agromyzid collections. Spencer’s book remains the primary identification tool for North European Agromyzidae. Interest in Finnish agromyzids has remained sporadic. The Finnish species were last listed by Hackman (1980).

**Number of species:**

World: 3004 spp. (M. Černý & M. von Tschirnhaus, pers. comm., March 2014)

Europe: 914 species (von Tschirnhaus, pers. comm., March 2014)

Finland: 279–280 species

Faunistic knowledge level in Finland: average

## Checklist

suborder Brachycera Macquart, 1834

clade Eremoneura Lameere, 1906

clade Cyclorrhapha Brauer, 1863

infraorder Schizophora Becher, 1882

clade Muscaria Enderlein, 1936

parvorder Acalyptratae Macquart, 1835

superfamily Opomyzoidea Newman, 1834

**AGROMYZIDAE** Fallén, 1823

AGROMYZINAE Fallén, 1823

***AGROMYZA*** Fallén, 1810

*Agromyza abiens* Zetterstedt, 1848

*Agromyza albipennis* Meigen, 1830

*Agromyza albitarsis* Meigen, 1830

*Agromyza alnibetulae* Hendel, 1931

*Agromyza alnivora* Spencer, 1969

*Agromyza alunulata* (Hendel, 1931)

*Agromyza ambigua* Fallén, 1823

= *niveipennis* Zetterstedt, 1848

*Agromyza anthracina* Meigen, 1830

= *freyi* Hendel, 1931

*Agromyza bicaudata* (Hendel, 1931)

*Agromyza cinerascens* Macquart, 1835

*Agromyza demeijerei* Hendel, 1920

*Agromyza erythrocephala* Hendel, 1920

*Agromyza flaviceps* Fallén, 1823

*Agromyza graminicola* Hendel, 1931

*Agromyza idaeiana* Hardy, 1853

= *potentillae* (Kaltenbach, 1864)

= *spiraeae* Kaltenbach, 1867

= *stackelbergi* (Frey, 1946)

*Agromyza igniceps* Hendel, 1920

= *lathyri* misid.

*Agromyza lapponica* Hendel, 1931

*Agromyza lucida* Hendel, 1920

*Agromyza luteitarsis* (Rondani, 1875)

= *intermittens* misid.

*Agromyza marionae* Griffiths, 1963

= *alandensis* Spencer, 1976

*Agromyza mobilis* Meigen, 1830

*Agromyza nana* Meigen, 1830

*Agromyza nigrella* (Rondani, 1875)

*Agromyza nigrescens* Hendel, 1920

*Agromyza nigripes* Meigen, 1830

*Agromyza nigrociliata* Hendel, 1931

*Agromyza orobi* Hendel, 1920

*Agromyza phragmitidis* Hendel, 1922

*Agromyza pseudoreptans* Nowakowski, 1967

= *urticae* Nowakowski, 1964 preocc.

*Agromyza quadriseta* Zlobin, 2001

*Agromyza reptans* Fallén, 1823

*Agromyza rufipes* (Meigen, 1830)

*Agromyza salicina* Hendel, 1922

*Agromyza sulfuriceps* Strobl, 1898

*Agromyza vicifoliae* Hering, 1932

***HEXOMYZA*** Enderlein, 1936

*Hexomyza schineri* (Giraud, 1861)

*Hexomyza simplicoides* (Hendel, 1920)

***MELANAGROMYZA*** Hendel, 1920

*Melanagromyza aenea* (Meigen, 1830)

*Melanagromyza aeneoventris* (Fallén, 1823)

*Melanagromyza angeliciphaga* Spencer, 1969

*Melanagromyza chaerophylli* Spencer, 1969

*Melanagromyza lappae* (Loew, 1850)

*Melanagromyza nigrissima* Spencer, 1976

*Melanagromyza oligophaga* Spender, 1990

*Melanagromyza pubescens* Hendel, 1923

*Melanagromyza submetallescens* Spencer, 1966

***OPHIOMYIA*** Braschnikov, 1897

*Ophiomyia beckeri* (Hendel, 1923)

= *goniaea* (Hendel, 1931)

*Ophiomyia cunctata* (Hendel, 1920)

*Ophiomyia curvipalpis* (Zetterstedt, 1848)

*Ophiomyia fennoniensis* Spencer, 1976

?= *eucodonus* Hering, 1960

= *melandryi* misid.

*Ophiomyia heringi* Starý, 1930

= *penicillata* misid.

*Ophiomyia labiatarum* Hering, 1937

= *persimilis* misid.

*Ophiomyia longilingua* (Hendel, 1920)

= *rostrata* misid.

*Ophiomyia maura* (Meigen, 1838)

*Ophiomyia nasuta* (Melander, 1913)

*Ophiomyia orbiculata* (Hendel, 1931)

= *hexachaeta* (Hendel, 1931)

= *nostradamus* (Hering, 1933)

= *paracelsus* (Hering, 1933)

*Ophiomyia pinguis* (Fallén, 1820)

*Ophiomyia pulicaria* (Meigen, 1830)

*Ophiomyia ranunculicaulis* Hering, 1949

*Ophiomyia vitiosa* Spencer, 1964

?= *alliariae* Hering, 1954

PHYTOMYZINAE Fallén, 1823

***AMAUROMYZA*** Hendel, 1931

**sg. *Amauromyza*** Hendel, 1931

*Amauromyza morionella* (Zetterstedt, 1848)

**sg. *Cephalomyza*** Hendel, 1931

= ***Trilobomyza*** Hendel, 1931

*Amauromyza chenopodivora* Spencer, 1971

= *abnormalis* misid.

*Amauromyza flavifrons* (Meigen, 1830)

*Amauromyza gyrans* (Fallén, 1823)

*Amauromyza karli* (Hendel, 1927)

= *Dizygomyza facialis* Frey, 1941 nom. nudum

*Amauromyza labiatarum* (Hendel, 1920)

*Amauromyza luteiceps* (Hendel, 1920)

*Amauromyza monfalconensis* (Strobl, 1909)

***AULAGROMYZA*** Enderlein, 1936

= ***Paraphytomyza*** Enderlein, 1936

*Aulagromyza buhri* (de Meijere, 1938)

*Aulagromyza fulvicornis* (Hendel, 1935)

*Aulagromyza hendeliana* (Hering, 1926)

*Aulagromyza heringii* (Hengel, 1920)

*Aulagromyza lucens* (de Meijere, 1924)

*Aulagromyza luteoscutellata* (de Meijere, 1924)

= *xylostei* misid.

*Aulagromyza similis* (Brischke, 1880)

*Aulagromyza tremulae* (Hering 1957)

*Aulagromyza tridentata* (Loew, 1858)

*Aulagromyza trivittata* (Loew, 1873)

***CALYCOMYZA*** Hendel, 1931

*Calycomyza artemisiae* (Kaltenbach, 1856)

*Calycomyza subapproximata* (Sasakawa, 1955)

***CERODONTHA*** Rondani, 1861

**sg. *Butomomyza*** Nowakowski, 1967

*Cerodontha caricivora* (Groschke, 1954)

*Cerodontha eucaricis* Nowakowski, 1967

*Cerodontha rohdendorfi* Nowakowski, 1967

*Cerodontha scirpi* (Karl, 1926)

= *scutellaris* misid.

= *semiposticata* misid.

*Cerodontha staryi* (Starý, 1930)

**sg. *Cerodontha*** Rondani, 1861

*Cerodontha affinis* (Fallén, 1823)

*Cerodontha denticornis* (Panzer, 1806)

*Cerodontha fulvipes* (Meigen, 1830)

= *femoralis* (Meigen, 1838)

*Cerodontha hennigi* Nowakowski, 1967

= *lateralis* (Zetterstedt, 1848) preocc.

*Cerodontha stackelbergi* Nowakowski, 1972

**sg. *Dizygomyza*** Hendel, 1920

*Cerodontha bimaculata* (Meigen, 1830)

*Cerodontha fasciata* (Strobl, 1880)

*Cerodontha ireos* (Robineau-Desvoidy, 1851)

*Cerodontha luctuosa* (Meigen, 1830)

*Cerodontha morosa* (Meigen, 1830)

**sg. *Icteromyza*** Hendel, 1931

*Cerodontha bohemani* (Rydén, 1951)

*Cerodontha capitata* (Zetterstedt, 1848)

*Cerodontha churchillensis* Spencer, 1969

*Cerodontha geniculata* (Fallén, 1823)

*Cerodontha lineella* (Zetterstedt, 1838)

**sg. *Phytagromyza*** Hendel, 1920

*Cerodontha flavocingulata* (Strobl, 1909)

= *storai* (Frey, 1946)

**sg. *Poemyza*** Hendel, 1931

*Cerodontha atra* (Meigen, 1830)

*Cerodontha calamagrostidis* Nowakowski, 1967

= *spenceri* Nowakowski, 1967

= *tschirnhausi* Nowakowski, 1973

*Cerodontha calosoma* (Hendel, 1931)

*Cerodontha imbuta* (Meigen, 1838)

= *deschampsiae* (Spencer, 1957)

*Cerodontha incisa* (Meigen, 1830)

*Cerodontha lateralis* (Macquart, 1835)

*Cerodontha muscina* (Meigen, 1830)

*Cerodontha phragmitidis* Nowakowski, 1967

*Cerodontha pygmaea* (Meigen, 1830)

*Cerodontha pygmella* (Hendel, 1931)

= *lapplandica* (Rydén, 1956)

*Cerodontha spencerae* Zlobin, 1993

= *inconspicua* auct. nec (Malloch, 1914)

*Cerodontha thunebergi* Nowakowski, 1967

**sg. *Xenophytomyza*** Frey, 1946

*Cerodontha atronitens* (Hendel, 1920)

*Cerodontha biseta* (Hendel, 1920)

= *crassinervis* (Frey, 1946)

*Cerodontha venturii* Nowakowski, 1967

***CHROMATOMYIA*** Hardy, 1849

*Chromatomyia asteris* (Hendel, 1934)

*Chromatomyia ciliata* (Hendel, 1935)

*Chromatomyia farfarella* (Hendel, 1935)

*Chromatomyia fuscula* (Zetterstedt, 1838)

*Chromatomyia glacialis* (Griffiths, 1964)

*Chromatomyia horticola* (Goureau, 1851)

*Chromatomyia isicae* (Hering, 1962)

*Chromatomyia linnaeae* Griffiths, 1974

*Chromatomyia lonicerae* (Robineau-Desvoidy, 1851)

= *xylostei* (Kaltenbach, 1862) preocc.

*Chromatomyia luzulae* (Hering, 1924)

*Chromatomyia milii* (Kaltenbach, 1864)

*Chromatomyia nigra* (Meigen, 1830)

*Chromatomyia norwegica* (Rydén, 1957)

*Chromatomyia ochracea* (Hendel, 1920)

*Chromatomyia opacella* (Hendel, 1935)

*Chromatomyia periclymeni* (Hendel, 1922)

*Chromatomyia primulae* (Robineau-Desvoidy, 1851)

*Chromatomyia ramosa* (Hendel, 1923)

= *nigriventris* (Hendel, 1935)

*Chromatomyia styriaca* Griffiths, 1980

*Chromatomyia syngenesiae* Hardy, 1849

***GALIOMYZA*** Spencer, 1981

*Galiomyza morio* (Brischke, 1880)

***LIRIOMYZA*** Mik, 1894

*Liriomyza amoena* (Meigen, 1830)

*Liriomyza angulicornis* (Malloch, 1918)

= *triglochinae* Hendel, 1931

*Liriomyza approximata* (Hendel, 1920)

*Liriomyza artemisicola* de Meijere, 1924

*Liriomyza bryoniae* (Kaltenbach, 1858)

*Liriomyza buhri* Hering, 1937

= *adolescens* Frey, 1946

*Liriomyza bulbata* Hendel, 1931

*Liriomyza canescens* Spencer, 1976

= *graminicola* misid.

*Liriomyza cannabis* Hendel, 1931

*Liriomyza congesta* (Becker, 1903)

= *minima* Hendel, 1931

= *parva* Hendel, 1931

*Liriomyza demeijerei* Hering, 1930

*Liriomyza equiseti* de Meijere, 1924

*Liriomyza eupatorii* (Kaltenbach, 1873)

= *orbitella* Hendel, 1931

*Liriomyza flaveola* (Fallén, 1823)

*Liriomyza flavopicta* Hendel, 1931

*Liriomyza freyella* Spencer, 1976

*Liriomyza hieracii* (Kaltenbach, 1862)

*Liriomyza infuscata* Hering, 1926

*Liriomyza lutea* (Meigen, 1830)

*Liriomyza occipitalis* Hendel, 1931

*Liriomyza orbona* (Meigen, 1830)

*Liriomyza pedestris* Hendel, 1931

*Liriomyza pseudopygmina* (Hering, 1933)

*Liriomyza ptarmicae* de Meijere, 1925

*Liriomyza pusilla* (Meigen, 1830)

= *fasciola* (Meigen, 1838)

*Liriomyza pusio* (Meigen, 1830)

= *graminicola* de Meijere, 1924

= *breviseta* Frey, 1946

*Liriomyza richteri* Hering, 1927

*Liriomyza sonchi* Hendel, 1931

*Liriomyza strigata* (Meigen, 1830)

= *pumila* (Meigen, 1830)

*Liriomyza tanaceti* de Meijere, 1924

*Liriomyza taraxaci* Hering, 1927

*Liriomyza valerianae* Hendel, 1932

*Liriomyza virgo* (Zetterstedt, 1838)

*Liriomyza virgula* Frey, 1946

*Liriomyza wachtlii* Hendel, 1920

***METOPOMYZA*** Enderlein, 1936

*Metopomyza flavonotata* (Haliday, 1833)

*Metopomyza interfrontalis* (Melander, 1913)

= *xanthaspida* (Hendel, 1920)

*Metopomyza scutellata* (Fallén, 1823)

= *flavoscutellaris* misid.

*Metopomyza xanthaspioides* (Frey, 1946)

*Metopomyza xanthaspis* (Loew, 1858)

***NAPOMYZA*** Westwood, 1840

*Napomyza achilleanella* von Tschirnhaus, 1992

*Napomyza bellidis* Griffiths, 1967

*Napomyza carotae* Spencer, 1966

*Napomyza cichorii* Spencer, 1966

*Napomyza elegans* (Meigen, 1830)

*Napomyza hirticornis* (Hendel, 1932)

*Napomyza lateralis* (Fallén, 1823)

*Napomyza maritima* von Tschirnhaus, 1981

*Napomyza merita* Zlobin, 1993

*Napomyza plumea* Spencer, 1969

***NEMORIMYZA*** Frey, 1946

*Nemorimyza posticata* (Meigen, 1830)

***PHYTOBIA*** Lioy, 1864

= ***Dendromyza*** Hendel, 1931

*Phytobia aucupariae* (Kangas, 1949)

*Phytobia cambii* (Hendel, 1931)

= *betulae* (Kangas, 1935)

= *tremulae* (Kangas, 1949)

*Phytobia mallochi* (Hendel, 1924)

***PHYTOLIRIOMYZA*** Hendel, 1931

*Phytoliriomyza arctica* (Lundbeck, 1901)

*Phytoliriomyza dorsata* (Siebke, 1863)

= *Liriomyza fasciata* misid.

= *pectoralis* misid.

*Phytoliriomyza hilarella* (Zetterstedt, 1848)

*Phytoliriomyza melampyga* (Loew, 1869)

*Phytoliriomyza mikii* (Strobl, 1898)

*Phytoliriomyza ornata* (Meigen, 1830)

= *elegantula* (Zetterstedt, 1848)

*Phytoliriomyza perpusilla* (Meigen, 1830)

***PHYTOMYZA*** Fallén, 1810

*Phytomyza abdominalis* Zetterstedt, 1848

= *gentianae* misid.

*Phytomyza aconitophila* Hendel, 1927

*Phytomyza actaeae* Hendel, 1922

*Phytomyza adjuncta* Hering, 1928

*Phytomyza albiceps* Meigen, 1830

= *rydeniana* Hering, 1949

*Phytomyza albipennis* Fallén, 1823

*Phytomyza anemones* Hering, 1925

*Phytomyza angelicae* Kaltenbach, 1872

*Phytomyza angelicastri* Hering, 1932

*Phytomyza aquilegiae* Hardy, 1849

*Phytomyza aquilonia* Frey, 1946

*Phytomyza artemisivora* Spencer, 1971

*Phytomyza buhriella* Spencer, 1969

*Phytomyza calthophila* Hering, 1931

*Phytomyza campanulae* Hendel, 1920

*Phytomyza chaerophylli* Kaltenbach, 1856

= *anthrisci* Hendel, 1924

= *carvi* Hering, 1931

*Phytomyza cirsii* Hendel, 1923

= *cirsicola* Hendel, 1927

*Phytomyza continua* Hendel, 1920

= *polyarthrocera* Frey, 1946

*Phytomyza crassiseta* Zetterstedt, 1860

*Phytomyza dasyops* Hendel, 1920

*Phytomyza diversicornis* Hendel, 1927

*Phytomyza enigmoides* Hering, 1937

= *enigma* Hering, 1936 preocc.

*Phytomyza erigerophila* Hering, 1927

*Phytomyza eumorpha* Frey, 1946

*Phytomyza evanescens* Hendel, 1920

= *calthivora* misid.

= *opaca* misid.

*Phytomyza fallaciosa* Brischke, 1880

= *pseudohellebori* Hendel, 1920

= *bonsdorfi* Hendel, 1935

*Phytomyza flavicornis* Fallén, 1823

*Phytomyza flavofemorata* Strobl, 1893

= *distantipila* Frey, 1950

= *aristata* misid.

= *pratensis* de Meijere, 1926

*Phytomyza glabra* Hendel, 1935

*Phytomyza glechomae* Kaltenbach, 1862

? *Phytomyza gymnostoma* Loew, 1858

*Phytomyza hellebori* Kaltenbach, 1872

*Phytomyza hendeli* Hering, 1923

*Phytomyza heracleana* Hering, 1937

*Phytomyza hirsuta* Spencer, 1976

*Phytomyza isais* Hering, 1936

*Phytomyza krygeri* Hering, 1949

*Phytomyza lappae* Goureau, 1851

*Phytomyza linguae* Lundqvist, 1947

*Phytomyza marginella* Fallén, 1823

*Phytomyza minuscula* Goureau, 1851

*Phytomyza murina* Hendel, 1935

*Phytomyza mylini* Hering, 1954

*Phytomyza nigrifemur* Hering, 1934

= *semitenella* Hendel, 1935

*Phytomyza nigripennis* Fallén, 1823

*Phytomyza nigritula* Zetterstedt, 1838

*Phytomyza notata* Meigen, 1830

*Phytomyza obscurella* Fallén, 1823

*Phytomyza pauliloewii* Hendel, 1920

*Phytomyza pimpinellae* Hendel, 1924

*Phytomyza plantaginis* Robineau-Desvoidy, 1851

*Phytomyza ptarmicae* Hering, 1937

*Phytomyza pubicornis* Hendel, 1920

*Phytomyza pullula* Zetterstedt, 1848

*Phytomyza ranunculi* (Schrank, 1803)

*Phytomyza ranunculivora* Hering, 1932

*Phytomyza rapunculi* Hendel, 1927

= *campanulivora* Spencer, 1971

*Phytomyza rhabdophora* Griffiths, 1964

*Phytomyza rostrata* Hering, 1934

*Phytomyza rufescens* von Roser, 1840

*Phytomyza rufipes* Meigen, 1830

*Phytomyza rydeni* Hering, 1934

*Phytomyza sedicola* Hering, 1924

*Phytomyza selini* Hering, 1922

*Phytomyza socia* Brischke, 1880

*Phytomyza soenderupi* Hering, 1941

*Phytomyza solidaginis* Hendel, 1920

*Phytomyza spinaciae* Hendel, 1935

= *affinis* misid.

*Phytomyza spoliata* Strobl, 1906

*Phytomyza spondylii* Robineau-Desvoidy, 1851

= *pastinacae* misid.

*Phytomyza subrostrata* Frey, 1946

*Phytomyza tanaceti* Hendel, 1923

*Phytomyza tenella* Meigen, 1830

*Phytomyza thysselini* Hendel, 1923

*Phytomyza trollii* Hering, 1930

*Phytomyza tussilaginis* Hendel, 1925

*Phytomyza varipes* Macquart, 1835

*Phytomyza virgaureae* Hering, 1926

*Phytomyza virosae* Pakalniškis, 2000

*Phytomyza wahlgreni* Rydén, 1944

= *robustella* misid.

***PSEUDONAPOMYZA*** Hendel, 1920

*Pseudonapomyza atra* (Meigen, 1830)

*Pseudonapomyza europaea* Spencer, 1973

***SELACHOPS*** Wahlberg, 1844

*Selachops flavocinctus* Wahlberg, 1844

## Excluded species

*Cerodontha silvatica* (Groschke, 1957) misidentification

*Dizygomyza facialis* Frey, 1941 nomen nudum

*Liriomyza huidobrensis* (Blanchard, 1926) imported

*Liriomyza sativae* Blanchard, 1938 imported

*Liriomyza trifolii* (Burgess in Comstock, 1880) imported

*Phytomyza atricornis* Meigen, 1838 nomen dubium

*Phytomyza brischkei* Hendel, 1922 misidentification

## Notes

***Agromyza nigrociliata***. First recorded from Finland by Thuneberg in Kaisila (1960). The species have since been found at several sites on the south coast (leg. L. Tiensuu, K. Winqvist & J. Kahanpää).

***Cerodontha pygmella***. First described from the Russian Far East, then redescribed from northern Sweden as *Cerodontha lapplandica* (Rydén) (synonymized by Zlobin 2001) and from Poland as *Cerodontha tatrica* Nowakowski (syn. Spencer 1976). The status of this species as distinct from *Cerodontha pygmaea* has been doubtful, but Zlobin (2001) pointed out that while *Cerodontha pygmella* and *Cerodontha pygmaea* are difficult to identify reliably as adults, the larvae are clearly different.

***Liriomyza pedestris*** Hendel, 1931 is a valid species according to Zlobin (2003). The Finnish material of the *Liriomyza flaveola*-group needs re-examination.

***Ophiomyia fennoniensis* Spencer** is probably a synonym of *Ophiomyia eucodonus* Hering. Likewise, ***Ophiomyia vitiosa*** Spencer is probably a synonym of *Ophiomyia alliariae* Hering, 1954 (M. von Tschirnhaus, pers. comm.)

***Phytomyza angelicae*** and ***Phytomyza aegopodii***. Spencer (1976) synonymized *Phytomyza aegopodii* Hendel, 1923 with *Phytomyza angelicae* Kaltenbach. Pakalniškis (2000) reinstated *Phytomyza aegopodii* as a valid species but opinions on its status are not unanimous. The Finnish material examined so far represents typical *Phytomyza angelicaesensu*Pakalniškis (2000).

***Phytomyza brischkei***. Recorded by Hackman (1980) from Finland, probably on the basis of Frey (1937). As Spencer (1976) points out, this is a misidentification.

***Phytomyza gymnostoma***. Recorded from Finland in Fauna Europaea (Martinez 2011), but I have been unable to trace the original records. Possibly imported to Finland with leeks or onions (*Allium*), its host plants. *Phytomyza gymnostoma* is an expanding species with Central Europe and has lately damaged crops of *Allium* species (Darvas et al. 1988).

***Phytomyza linguae***. Spencer (1976) synonymised this species with *Phytomyza ranunculivora* Hering. Pakalniškis (1997) reinstated it as a valid species. Both species occur in Finland: L. Tiensuu reared *Phytomyza ranunculivora* from *Ranunculus acer* and *Phytomyza linguae* from *Ranunculus lingua* from mines collected from Vehkalahti in 1972–3.
